# Dialect diversity and total factor productivity: Evidence from Chinese listed companies

**DOI:** 10.3389/fpsyg.2022.1017397

**Published:** 2022-11-11

**Authors:** Jiacai Xiong, Linghong Chen

**Affiliations:** School of Accounting, Jiangxi University of Finance and Economics, Nanchang, China

**Keywords:** dialect diversity, total factor productivity, psychological distance, innovation, element flow, human capital accumulation

## Abstract

Using a sample of Chinese listed companies over the 2007–2019 period, we examined the influence of dialect diversity on a firm's total factor productivity. We found that dialect diversity affects the psychological distance of interpersonal communication and significantly affects the firm's total factor productivity. The results are robust to a battery of tests based on different specifications. The relationship between dialect diversity and a firm's total factor productivity is more pronounced in state-owned enterprises, firms located in southern regions, and more capital-intensive firms. Furthermore, we demonstrated an innovative factor flow mechanism and a human capital accumulation mechanism through which dialect diversity inhibits total factor productivity. Overall, this paper provides new evidence and decision-making reference for coordinating the protection of dialect diversity and high-quality economic development.

## Introduction

Since the market-oriented reform and opening up in 1978, China has witnessed tremendous gross domestic product (GDP) growth from <$150 billion in 1978 to $17.7 trillion in 2021. However, China's growth has come mainly from a rising labor supply and rapid capital accumulation, which created significant pressure on its natural resources and the environment, and is unsustainable in the long run. Therefore, China has branched into productivity growth through several high-profile initiatives such as the “Made in China 2025” (Li, 2018). Krugman (1995) points out that “Productivity isn't everything, but in the long run, it is almost everything,” which illustrates the importance of productivity. The growth of productivity is considered the main driver of high-quality economic development. Thus, how to effectively improve the total factor productivity of enterprises attracts significant interest from researchers. However, the extant literature focuses on the characteristics of enterprises (Tian and Twite, 2011) and the level of an economic system (Wan and Zhang, 2018; Fu et al., 2021), and fewer papers discuss the influence of informal institutional factors on total factor productivity (TFP) of enterprises, especially for listed companies in China.

China is a multi-ethnic country where multiculturalism coexists, thus providing an ideal setting for studying non-institutional factors and total factor productivity. As the carrier of regional culture, language is not only a vehicle for people's expression and communication but also exerts a subtle influence on individual behavior (Chen, 2013). In recent years, some literature also uses language or dialect as proxy variables of culture (Bian et al., 2019). However, the area of studies related to the economic influence of dialect has developed relatively late and is still in its infancy. The longstanding and heated debates over dialect diversity focus mainly on its relationship with economic performance (Zhu and Grigoriadis, 2022), FDI (Feng et al., 2021), sustainable trade development (Liu et al., 2022), or urban size (Ding et al., 2021), among others; few studies have focused on the effect of multiculturalism on TFP based on micro-enterprise data. Therefore, the current work focuses on one dimension of culture, i.e., language, investigates the effects of dialect diversity on a firm's TFP, and explores the mechanism between them.

Dialect diversity is the accumulation of culture and social psychology, which is the first language of most people. Dialects contain the emotions of people and the country and can better and more directly express thoughts and feelings. Therefore, dialect diversity can influence psychological distance in interpersonal interactions. Psychology-related studies show that managers' perceived similarities affect their attraction (Huston and Levinger, 1978). Compared to dissimilar individuals, similar individuals have more mental dependence and identification, higher perceived similarity, stronger mutual attraction, and more accessible communication, and thus more likely to interact with each other (McPherson et al., 2001). In China, there is a prominent vernacular bond among interpersonal interactions, and the vernacular bias leads to a higher similarity between people who speak the same dialect, increasing the level of trust and facilitating communication. Dialect diversity becomes an extrinsic signal of perceived similarity. However, most of the existing studies focus on the identity effects of dialects (Falck et al., 2012) and the fact that dialects affect the sustainable development of trade (Guiso et al., 2009; Liu et al., 2022), and the cultural differences behind the dialect diversity (Chen, 2013). Few studies have explored the effects of dialect diversity on the TFP of firms from a psychological distance perspective.

To empirically solve the above challenges, we used a sample that contains all non-financial A-share listed firms in China between 2007 and 2019. The main variable used to measure TFP was calculated following previous studies such as Ackerberg et al. (2015), Levinsohn and Petrin (2003), and Wooldridge (2009). In addition, we followed Xu et al. (2015) and Lei et al. (2022) and measured regional cultural diversity through dialect diversity. Based on the analysis of the psychological distance, our empirical results show that regional cultural diversity, represented by dialect diversity, substantially decreased TFP by 3.43%. Specifically, the TFP of firms located in regions with higher dialect diversity is less than those in other regions. Moreover, the results are robust to several robust checks, including two-stage instrumental variable regression to solve the endogenous problem, alternative measures of TFP, and controlling the high-order fixed effect of industry and year.

Although dialect diversity significantly negatively affects firm TFP, this effect exhibits heterogeneity in different dimensions. Based on the subsample test, we further documented that the negative effect of dialect diversity on TFP is particularly pronounced in state-owned enterprises, capital-intensive firms, and firms located in the southern region. After documenting that dialect diversity decreases TFP, we proposed and verified three mechanisms through which dialect diversity could hamper innovation, factor flow, and human capital accumulation.

This study contributes to the literature in several ways. First, our results draw attention to the influence of cultural diversity on micro-enterprise. Previous research on informal institutions primarily focuses on religiosity, gambling, or social trust (e.g., Bjørnskov, 2012; Boone et al., 2013; Wang, 2021; Xie and Wang, 2022). Our findings suggest that it is helpful for firms to focus on the effects of dialect diversity in their localities. As an informal institution, dialect diversity in a region where a firm is located contributes to curbing corporate total factor productivity. Furthermore, we examined the inhibition effect of enterprises in different regions, industries, ownership types, capital intensity, and social trust, which provided evidence of dialect diversity's heterogeneous effect.

Second, we discussed the negative relationship between dialect diversity, a critical informal institution, and the TFP of enterprises, which enriches the research on the factors of TFP. Although the determinants of TFP are extensively discussed in previous works (Hsieh and Klenow, 2009; Xiao et al., 2022), how dialect diversity affects the TFP of listed enterprises has remained unclear. Hence, our research seeks to provide a deeper understanding of how dialect diversity affects TFP and enriches the studies on the relationship between dialect diversity and the TFP of enterprises. In addition, our findings also suggest that innovation mechanisms, factor flow mechanisms, and human capital accumulation mechanisms through which dialect diversity affects firm TFP, which enrich our understanding of the underlying micro-mechanisms that dialect diversity affects by linking our finding to the literature on determinants of firm-level TFP.

The structure of this study is as follows. Section “Literature review and hypothesis development” reviews related literature and develops the hypothesis, section “Research design” discusses the research methodology, section “Empirical results” reports the results of the empirical analysis and provides robustness checks, and section “Further analyses” presents further analyses. Finally, section “Conclusion” offers the conclusion.

## Literature review and hypothesis development

### Literature review

#### Literature on dialect diversity

Due to a relatively weak legal system and imperfect formal institutions (Allen et al., 2005; Pistor and Xu, 2005), China is still a guanxi (relationship)-based society, which is affected mainly by informal institutions (Xin and Pearce, 1996; Li, 2003), and the research about culture as a non-institutional factor is insufficient. As an informal institution, culture can influence individuals' personality, cognition, and communication styles (DiMaggio, 1997; Wang, 2021; Xie and Wang, 2022) and even shape human economic behavior (Chen, 2013). Language is the medium of communication and the carrier of culture, which is a crucial indicator for measuring cultural diversity (Gong et al., 2011; Falck et al., 2012; Bian et al., 2019). At present, the research on the influence of dialect diversity on the cultural economy can be divided into three categories.

The subject of the first category of research is differences in cultural concepts behind dialects. Chen (2013) pointed out that the more significant the difference in language, the more considerable the difference in cultural concepts. Therefore, cultural differences caused by the diversity of dialects hinder the spread of institutions and hence affect the diffusion of technology and production factors (Spolaore and Wacziarg, 2009; Spolaore, 2012; Ding et al., 2021). Moreover, cultural differences lead to regional cultural diversity and affect the divided areas' economic performance (Zhu and Grigoriadis, 2022).

The second type of literature focuses on the effects of dialect on transaction costs. Dialect differences tend to make people distrustful, resulting in increased transaction costs (Wang and Ruan, 2019), which is not conducive to teamwork and innovation success. It also increases the transaction cost of enterprise innovation outsourcing and decreases the innovation investment of enterprises (Milliken and Martins, 1996; Zhang and Wang, 2022). However, the presence of a common language reduces the cost of information search when hedging economic risks (Egger and Lassmann, 2015), thus reducing transaction costs in international trade (Melitz, 2008).

The third type of literature emphasizes the identity recognition function of dialects. Dialects have an identity effect; thus, individuals using the same dialect have a higher level of identity (Gumperz, 1982). Dialect diversity influences business innovation through cultural identity (Wang et al., 2022). The identity effect of dialects also affects labor income (Falck et al., 2018), and people speaking local dialects are likely to earn higher income in the labor market (Chen et al., 2014). In addition, it was found that the identity effect of the dialect also affects the behavior of managers and entrepreneurs. For example, the dialect of the CEO and the general manager unanimously reduced the agency cost of the company (Bian et al., 2019).

Although numerous studies have attempted to study the impact of dialect on the economy, most of them have concentrated solely on the macroeconomy. Moreover, few studies have explicitly examined the influence of dialect diversity on enterprise total factor productivity based on psychological distance.

#### Literature on TFP

At the micro level, previous studies found that the source of TFP is shifting resource allocation from less efficient to more efficient enterprises (Hall and Jones, 1999; Caselli, 2005; Bartelsman et al., 2013). The improvement of capital and labor resource allocation efficiency and talent introduction can promote the improvement of the TFP of enterprises (Hsieh and Klenow, 2009). However, the financial friction caused by resource misallocation decreases TFP (Wang et al., 2021). Moreover, R and D spillover, R and D activities, technology development, and transformation investment can significantly improve enterprise TFP, while financial constraints significantly reduce the enterprise TFP (Caggese and Cuñat, 2013; Xiao et al., 2022). The literature also found a strong coupling between the system and economic development at the macro level. For economies at a particular stage of development, TFP enhancement and economic development can only be promoted by adopting compatible institutions (Acemoglu, 2003; Glaeser et al., 2004). Besides, financial friction leads to resource mismatch and results in the loss of TFP (Wang et al., 2021).

However, most empirical works have focused on the influence of formal systems on TFP. We complement this research gap by focusing on China, the world's most multicultural country, and investigate the influence of dialect diversity on TFP.

### Hypothesis development

Language is representative of a nation and culture and affects psychological distance in interpersonal interactions (Xu et al., 2015). Different nationalities and cultures have different customs and habits, which also cause language barriers. However, shared cultural preferences close the psychological distance between people, making it easier for people to communicate with each other. As a cultural carrier, language is the primary tool for people to exchange information. Language differences not only create communication barriers but also reduce the similarity between members and affect the psychological distance of interpersonal interactions. It reduces the level of social trust and leads to weak social relationships and a lack of trust in interpersonal interactions (Pendakur and Pendakur, 2002).

First, the increased psychological distance between individuals reduces communication and collaboration efficiency and makes transactions and collaboration more costly. Dialect diversity increases the psychological distance in firms, affects communication and exchange, reduces economic interaction and cooperation, increases transaction costs (Wang and Ruan, 2019), and reduces the level of innovation in firms (Zhang and Wang, 2022), which hinders TFP.

Second, dialect diversity reduces the perceived similarity of individuals, causing fragmentation of social networks and indirectly raising the barriers to the integration of elements such as people and property. The more complex the linguistic environment of a region, the greater the cultural differences, the greater the psychological distance between individuals, and the greater the degree of fragmentation of social networks tends to be. As a result, the tendency for factors of production and technology to flow in will be significantly weaker since people prefer to work in regions with similar dialectal cultures and simple language environments (Falck et al., 2012; Ding et al., 2021).

Finally, dialect diversity is a symbol of social identity, and its identification effects create a psychological distance between members of different dialect groups. Linguistic similarities bring each other closer psychologically, while differences may be labeled as “not my kind,” creating “in-group preferences” and “out-group discrimination” (Tajfel, 1974; Hazen, 2001). Groups with different dialectal diversity have different dialectal identities, are prone to mutual distrust and disagreement, and reduce economic interactions and cooperation (Feng et al., 2021), thus creating an implicit barrier to increasing the TFP of firms.

The above analyses lead to the first testable hypothesis:

**H1:** *Dialect diversity has an inhibitory effect on TFP*.

In addition to the main research question, we intend to explore how dialect diversity affects TFP.

Technological progress and resource allocation optimization are critical paths to improving TFP (Perelman, 1995; Dong et al., 2021). Resource allocation allocates limited combinations of factors of production in more efficient sectors to achieve optimization of input factor structures, which improves TFP (Wang et al., 2021). Li and Meng (2014) pointed out that the labor force tends to flow relatively easily between regions with the same dialect, and dialect diversity hinders the flow of production factors (Ding et al., 2021). Falck et al. (2012) proposed that dialect diversity affects population mobility. When the linguistic environment is too complex, different cultures will lead to a lower level of social trust, thus hindering the mobility of labor factors. Dialect diversification reduces social trust between groups through identity effects, hinders the free flow of production factors, and thus inhibits TFP.

Innovation can increase a firm's productivity by enhancing its innovation capabilities and absorbing advanced external technologies (Xiao et al., 2022). However, studies show that dialect diversity inhibits business innovation. Wang et al. (2022) found that dialect diversity leads to communication barriers, hinders inter-group communication, discourages technology diffusion, and significantly inhibits firms' innovation. Furthermore, Hu et al. (2022) pointed out that dialect diversity contributes to cultural differences, which reduce mutual attraction and opportunities for cooperation and communication and hence reduce innovation input. As a result, dialect diversity increases transaction costs, creates cultural differences, and reduces firms' innovation inputs, decreasing TFP.

Based on this, we propose the following hypotheses.

**H2:** *Dialect diversity hinders the free flow of production factors and hence decreases TFP*.

**H3:** *Dialect diversity reduces innovation inputs and hence inhibits TFP*.

Furthermore, human capital accumulation plays a positive role in TFP (Salinas-Jiménez et al., 2006). Human capital has a knowledge effect and can either promote technological progress through active R and D innovation (Poncet et al., 2010) or boost technological innovation by learning, imitating, and mastering advanced technologies (Chang et al., 2019), thus increasing TFP. However, dialect diversity inhibits human capital accumulation. The more complex and diverse the dialects of a region, the less conducive it is for outsiders to master the local dialect, which indirectly raises the cost of environmental integration and hinders the cross-regional mobility of talents (Zhang and Wang, 2022). If a region has more dialects, it may imply the reluctance of people in this region to accept other dialects or learn foreign languages (Pendakur and Pendakur, 2002). Drummond (2013) also found that dialects severely affect English language learning ability, causing some students to drop out of school and preventing the residents from developing their abilities. In conclusion, dialect diversity prevents residents from improving their abilities, inhibits the accumulation of human capital, and thus reduces the TFP of enterprises.

As such, we propose the following hypothesis:

**H4:** *Dialect diversity is detrimental to regional human capital accumulation, inhibiting firms' TFP*.

## Research design

### Sample selection

Following Du et al. (2022) and Lei et al. (2022), our sample included Chinese A-share listed companies from 2007 to 2019. We started our sample period in 2007 because it was the year that China adopted the International Accounting Standard. The financial data were retrieved from the Wind and China Stock Market & Accounting Research (CSMAR) databases. The data on provinces and cities were obtained from the China Statistical Yearbook and the China City Statistical Yearbook. At the same time, data on the dialect diversity in the region where the firms were located was obtained from a dialect database constructed by Xu et al. (2015). Following Du et al. (2022) and Lei et al. (2022), we cleaned the sample as follows: (1) companies in the financial industries were removed; (2) samples with missing data were dropped; (3) ST and ^*^ST company samples were excluded; (4) continuous variables were winsorized at the levels of 1% and 99%. After the above screening, the whole sample consisted of 10,875 firm-year observations.

### Model specification and variable definition

Following Tian and Twite (2011) and Lei et al. (2022), we employed the following regression model to test whether dialect diversity affected TFP as predicted:


(1) $$\begin{array}{l} TFP_{it}=\alpha \;+\;\beta Diver_{it}\;+\;\sum Controls_{it}\;+\;Year\;+\;Industry\;+\;\varepsilon \end{array}$$

where *TFP*_*it*_ represents TFP of firm *i* in year *t*. *Diver*_*it*_ is dialect diversity index, ∑ *Control*_*it*_ represents control variables, ε is the error term, and α and β are the coefficients to be estimated. We controlled for industry and year-fixed effects to mitigate industry differences and economic fluctuation. All the standard errors in the regression were clustered at the firm level.

#### Dependent variables

There are many methods to calculate TFP, such as Levinsohn and Petrin (2003) (LP for short), Olley and Pakes (1996) (OP for short), and Ackerberg et al. (2015) (ACF for short). However, compared with other methods, the ACF can overcome the endogenous function and the time-varying parameters and measure the TFP more accurately (Bournakis and Mallick, 2018). Hence, we used the ACF method to estimate TFP (henceforth *TFP_ACF*), which was estimated by the following equation:


(2) $$\begin{array}{l} LnY_{it}=\beta_{0}+\beta_{k}LnK_{it}+\beta_{l}LnL_{it}+\beta_{m}LnM_{it}+\beta_{a}Age_{it}\\ +\beta_{s}Soe_{it}+Year+Industry+\varepsilon \end{array}$$

where *Y* is the output variable, proxied by operating income; *L* is the labor input, measured by the number of employees; *K* is the capital input, measured by net fixed assets; *M* is the intermediate input, expressed by the cash for buying goods and receiving services. *Age* is the enterprise age; *Soe* is an indicator variable equal to one for state-owned enterprises and zero otherwise. *Year* and *Industry* are year and industry fixed effects, respectively. Referring to the existing literature (Yu and Qi, 2022), we also used the LP and WRDG methods as alternative estimates in the robustness check.

#### Explanatory variables

The core explanatory variable *Diver*_*i*_ was used to measure the dialectal diversity of the city. The key independent variable of interest was the diversity level of dialects in each city. This indicator was obtained from Xu et al. (2015); Wang J. et al. (2021), and Lei et al. (2022), and it measured the dialect diversity of city *j* by considering the number of people who used different dialects. *Diver*_*i*_ was calculated as equation (3):


(3) $$\begin{array}{l} {Diver}_{it}\;=\;1-\;\sum_{j=1}^{N}S_{ij}^{2} \end{array}$$

where *S*_*ij*_ refers to the proportion of the population speaking dialect *j* in city *i*, and *N* is the number of dialects spoken in the city. The value range of *Diver*_*it*_ was from 0 to 1.5. The larger the value, the higher the degree of dialect diversity it represents.

#### Control variables

Following Kong et al. (2020), Wang J. et al. (2021), and Lei et al. (2022), we also included two sets of control variables related to firm-specific and region-specific characteristics, respectively. The firm-specific variables included (1) firm size (*Size*), which is the natural logarithm of the total assets of the enterprise; (2) debt-to-asset ratio (*Lev*), which refers to the ratio of total liabilities to total assets; (3) return on assets (*Roa*), which is the ratio of the net income over total assets; (4) main business revenue growth (*Growth*), which refers to the difference between current-year main business revenue and prior-year main business revenue to prior-year sales; and (5) ownership concentration (*Top10*), which pertains to the shareholding ratio of the top 10 shareholders.

The region-specific variables include economic growth (*GDP_city*), which represented the city's actual per capita GDP, and fixed-asset investment (Fixedcapital_GDP), which is the first 3-year average of the proportion of fixed asset investment to GDP in each province. Finally, we controlled for year- and industry-fixed effects.

## Empirical results

### Descriptive statistics of variables

Table 1 presents descriptive statistics of the main variables. The mean of *TFP_ACF* was 9.502, with a maximum value of 12.09 and a minimum value of 7.806, indicating that *TFP_ACF* varied largely among firms. Moreover, the differences between Diver's maximum and minimum values were significant, implying that regional cultural diversity varies largely among cities, providing a suitable setting for our study. The results of the remaining control variables were also in line with expectations and were not required to be repeated.

**Table 1:** Summary statistics.

**Variables**	***N***	**Mean**	**SD**	**Median**	**Min**	**Max**
*TFP_ACF*	10,875	9.502	0.863	9.388	7.806	12.090
*Diver*	10,875	0.226	0.194	0.228	0.002	0.653
*GDP_city*	10,875	8.231	3.916	7.899	1.428	18.960
*Fixedcapital_GDP*	10,875	0.558	0.243	0.557	0.172	1.165
*Size*	10,875	22.210	1.317	22.020	19.590	26.380
*Lev*	10,875	0.466	0.209	0.468	0.053	0.940
*Roe*	10,875	0.060	0.118	0.065	−0.701	0.329
*Growth*	10,875	0.455	1.333	0.131	−0.733	9.639
*Top10Hold*	10,875	56.640	15.560	57.260	22.410	96.29

This table reports the descriptive statistics of main variables. Specifically, it includes the mean, standard deviation (SD), median, minimum (Min), and maximum (Max) distributions.

### Results of the multivariate regression

Table 2 reveals dialect diversification's influence on enterprises' TFP based on Equation (1). Column (1) reports the results without control variables, and column (2) is the regression results with control variables. The results in column (1) show that dialect diversification is negatively related to the TFP of a firm when only the industry- and annual-fixed effects are controlled. The estimated coefficient of *Diver* was −0.303 (SE value = 0.085), which was significant at the level of 1%. After adding the control variables at the company and regional levels, the estimated coefficient of *Diver* in column (2) was still significantly positive at the level of 1%. The above results were not only statistically significant but also economically significant. Compared with the sample average and median, TFP decreased by 3.39% (=0.322/9.502) and 3.43% (=0.322/9.388), respectively. Baseline regression results show that dialect diversification significantly and negatively affected TFP, suggesting that **Hypothesis 1** in this paper cannot be rejected.

**Table 2:** Results for the effect of dialect diversity on TFP.

	**(1)**	**(2)**
	***TFP_ACF***	***TFP_ACF***
*Diver*	−0.303***	−0.322***
	(0.085)	(0.074)
*GDP_city*		0.015***
		(0.005)
*Fixedcapital_GDP*		−0.120
		(0.077)
*Size*		0.180***
		(0.013)
*Lev*		0.519***
		(0.084)
*Roe*		1.364***
		(0.101)
*Growth*		0.021***
		(0.008)
*Top10Hold*		0.003***
		(0.001)
*Year FE*	YES	YES
*Industry FE*	YES	YES
*N*	10,875	10,875
Adj. *R*^2^	0.243	0.407

This table reports the impact of dialect diversity on firm's total factor productivity. The dependent variable is total factor productivity (TFP_ACF) and calculated using the ACF method. Diver is the dialectal diversity indicator taken from Xu et al. (2015) and Wang J. et al. (2021) to measure the dialectal diversity of the city. Column (1) reports the regression without control variables, and column (2) adds control variables. The definition of all variables can be seen in section “Model specification and variable definition.” All regressions control for Industry FE and Year FE and T-statistics are provided in parentheses.

^***^, ^**^, and ^*^ indicate significance at the 1, 5, and 10% levels, respectively. Standard errors are clustered at the firm level.

When the coefficients of control variables are significant, consistent with previous studies, coefficients such as *Size, Growth*, and *Top10Hold* are positive and significant. According to Sleuwaegen and Goedhuys (2002), the larger the enterprise (*Size* is large), the stronger its ability to purchase advanced equipment and attract technical personnel, and the more capital to invest in R&D activities, thereby improving the TFP. The growth of an enterprise (*Growth*) is significantly and positively correlated with TFP, indicating that the better the growth of an enterprise, the greater the investment expenditure, and the faster the speed of product and services and technology updates further to improve TFP (Palia and Lichtenberg, 1999). In addition, our finding on ownership concentration (*Top10Hold*) was also consistent with those in Holderness and Sheehan (1988) in that equity concentration is positively related to innovation. The improvement of innovation ability promotes enterprise efficiency and further improves TFP.

### Robustness test

#### Two-stage instrumental variable regression

Following Wang J. et al. (2021), we employed the Chinese topographic fluctuation index (Slope) as the instrumental variable to solve the potential endogenous problem and estimate it using the two-stage least squares method (2SLS). On the one hand, the geographical factors were closely linked to dialect formation; that is, the more complex the terrain, the more geographic obstacles in the region. Then, it is more likely to be divided into different areas, producing various dialects. As a result, each dialect area will form a unique local culture. On the other hand, the TFP of enterprises would have difficulty affecting the natural condition of the terrain, especially in modern society. The increasing convenience of transportation and the continuous development of construction technology has gradually lessened the effect of terrain slope on enterprise business activities.

The 2SLS regression results are presented in Table 3. Column (1) shows that the relief degree of the land surface (*Slope*) was positively related to regional cultural diversity, suggesting that the greater the topography of an area, the more diverse the dialects of the area. Meanwhile, the values of the F statistic were 35 (far >10), which shows that the instrumental variable we designed was appropriate and no weak instrumental variable problem exists. Finally, column (2) shows that the relationship between regional cultural diversity and TFP was significantly negative at the 5% level in the second-stage regressions, and the absolute value of the coefficient was larger than the results of the baseline regression, which shows that dialect diversity had a significant and robust inhibitory effect on TFP.

**Table 3:** Regression results based on the instrumental variable method.

	**(1)**	**(2)**
	**First-stage regressions**	**Second-stage regressions**
	***Diver***	***TFP_ACF***
*Slope*	0.051***	
	(0.006)	
*Diver*		−0.823**
		(0.374)
*Control*	Yes	Yes
*Industry FE*	Yes	Yes
*Year FE*	Yes	Yes
*N*	10,825	10,875
*Adj. R^2^*	0.058	0.394

This table reports the results of the instrumental variable regression. Slope is the Chinese topographic fluctuation index. The definition of all other variables can be found in section “Model specification and variable definition.” A series of fixed effects are also included, and T-statistics are provided in parentheses.

^***^ and ^**^ indicate significance at the 1 and 5% levels, respectively. Standard errors are clustered at the firm level.

#### Alternative measure of TFP

Following Levinsohn and Petrin (2003) and Wooldridge (2009), we did a series of robustness tests using the LP and WRDG methods to measure firm productivity. The coefficient estimates obtained by the OLS can be biased; the LP method can overcome this drawback and better cope with simultaneity and sample selection problems (Zhang and Liu, 2017). The WRDG method was based on the GMM model, further improving the LP and ACF methods.

Table 4 presents the robustness checks using the LP and WRDG methods. The core explanatory variable *Divers*' coefficient was significantly negative in columns (1) and (2), indicating that the robustness regression results still support this paper's baseline conclusions.

**Table 4:** Robustness checks using an alternative TFP measure.

	**(1)**	**(2)**
	***TFP_LP***	***TFP_WRDG***
*Diver*	−0.151**	−0.139**
	(0.070)	(0.069)
*Control*	Yes	Yes
*Year FE*	Yes	Yes
*Industry FE*	Yes	Yes
*N*	10,875	10,869
*Adj. R^2^*	0.652	0.845

This table reports the results of the robustness checks measuring TFP using other methods. TFP_LP represents the TFP measured by the LP method, and TFP_WRDG represents the TFP measured by the WRDG method. The definition of all other variables can be found in section “Model specification and variable definition.” A series of fixed effects are also included and T-statistics are provided in parentheses.

The ^**^ symbol indicates the significance at the 5% level. Standard errors are clustered at the firm level.

## Further analyses

### Mechanism analyses

This part examines the intermediary effect of factor flow, human resource accumulation, and innovation. According to the relevant analysis in the second part, factor flow, the accumulation of human capital, and innovation investment influence the relationship between dialect diversity and TFP.

First, dialect diversity produces cultural segmentation in different regions. The more local dialect diversity, the lower the level of social trust would be, which raises the barriers to the integration of production factors and hinders the flow of labor and capital between different regions (Falck et al., 2012; Ding et al., 2021).

Second, dialect diversity is not conducive to outsiders mastering the local language; it hinders the introduction of advanced talents and knowledge and significantly affects the accumulation of local human capital, thereby affecting enterprises' TFP.

Finally, dialect diversity also decreases the TFP of enterprises through innovation input; the effects of innovation may be explained in at least two different ways. First, dialect diversity is not conducive to communication because it increases information asymmetry and transaction costs (Milliken and Martins, 1996), reducing enterprise innovation investment and inhibiting enterprise TFP (Xiao et al., 2022). Second, dialect diversity inhibits factor mobility and human capital accumulation in firms, leading to market segmentation and diminishing the scope for firms to be compensated for innovation inputs through economies of scale, thereby reducing firms' innovation investment (Foellmi and Zweimüller, 2006), which affects firms' TFP.

#### Verification of factor flow mechanism

Referring to Baron and Kenny (1986), the following mediation effect model was constructed to investigate whether dialect diversity can affect the TFP of enterprises through the factor flow mechanism.


(4) $$\begin{array}{l} {TFP}_{it}=\alpha +\beta {Diver}_{i}+\sum {Controls}_{it}+Year+Industry+\varepsilon \end{array}$$

(5) $$\begin{array}{l} {Flow}_{it}=\alpha +\beta {Diver}_{i}+\sum {Controls}_{it}+Year+Industry+\varepsilon \end{array}$$

(6) $$\begin{array}{l} \begin{array}{l} \begin{array}{l} TFP_{it}\;=\;\alpha +\beta_{1}Diver_{i}+\beta_{2}Flow_{it}+\sum^{\text{​}}Controls_{it}+Year\;\\ +Industry+\varepsilon \end{array} \end{array} \end{array}$$

*Flow*_*it*_ in formulas (5) and (6) represents factor flow, including capital and labor flows. Capital flow (*Klow*_*it*_) was measured using the logarithm of municipal fixed asset investment. Labor flow (*Llow*_*it*_) was measured by the proportion of the difference between the permanent resident population and the registered population. Other control variables remained the same as described above. The estimated results of the factor flow mechanism test are reported in Table 5.

**Table 5:** Results for mechanism analyses: factor flow.

**Panel A: Capital flow**
	**(1)**	**(2)**	**(3)**
	***TFP_ACF***	***Kflow***	***TFP_ACF***
*Diver*	−0.322***	−0.577***	−0.285***
	(0.074)	(0.082)	(0.075)
*Kflow*			0.063***
			(0.021)
*Controls*	Yes	Yes	Yes
*Year FE*	Yes	Yes	Yes
*Industry FE*	Yes	Yes	Yes
*N*	10,875	10,875	10,875
*Adj. R^2^*	0.407	0.371	0.409
**Panel B: Labor flow**
	**(1)**	**(2)**	**(3)**
	***TFP_ACF***	***Lflow***	***TFP_ACF***
*Diver*	−0.322***	−0.249***	−0.262***
	(0.074)	(0.019)	(0.077)
*Lflow*			0.241***
			(0.089)
*Controls*	Yes	Yes	Yes
*Year FE*	Yes	Yes	Yes
*Industry FE*	Yes	Yes	Yes
*N*	10,875	10,875	10,875
*Adj. R^2^*	0.407	0.544	0.409

This table presents the factor flow mechanism results between dialect diversity and TFP. Kflow is measured by the logarithm of municipal fixed asset investment. Lflow is measured by the proportion of the difference between the permanent resident population and the registered population in the registered population. Panel A reports the mechanism analyses results of capital flow, and Panel B reports the results of the mediation test for labor flow. The definition of all other variables can be seen in section “Model specification and variable definition.” A series of fixed effects are also included, and T-statistics are provided in parentheses.

The ^***^ symbol indicates the significance at the 1% level. Standard errors are clustered at the firm level.

Panel A of Table 5 shows the mechanism analyses results of capital flow. Column (2) presents the regression result of the model (5). It shows that the regression coefficient of capital flow (*Kflow*) on *Diver* was negative and significant at 1%, indicating that dialect diversification significantly suppressed capital inflow under the control of other unchanged variables. Column (3) presents the estimation result of the model (6). After the inclusion of the intermediary variable *Kflow*, the absolute value of the *Diver* coefficient was significantly smaller, and the coefficient of capital flow (*Kflow*) was significantly positive, indicating that dialect diversity hinders the free flow of capital, thereby inhibiting TFP.

Panel B of Table 5 shows the results of the mediation test for labor flow. Columns (2) and (3) indicate the regression of models (5) and (6), respectively. The coefficients *Diver* in column (2) was significantly negative. After including the intermediary variable *Lflow*, the absolute value of the *Diver* coefficient was significantly smaller in column (3), indicating that dialect diversity hindered the labor flow and thus decreased the TFP. The regression results in Table 5 indicate that dialect diversification can suppress the TFP of enterprises through the factor flow mechanism. Therefore, **Hypothesis 2** holds.

#### Verification of human capital accumulation mechanism

The following model was constructed to investigate whether dialect diversity can affect the TFP of enterprises through the human capital accumulation mechanism.


(7) $$\begin{array}{l} {TFP}_{it}\;=\alpha +\beta {Diver}_{i}+\sum {Controls}_{it}+Year+Industry+\varepsilon \end{array}$$

(8) $$\begin{array}{l} {Perstu}_{it}=\alpha +\beta {Diver}_{i}+\sum {Controls}_{it}+Year+Industry+\varepsilon \end{array}$$

(9) $$\begin{array}{l} \begin{array}{l} \begin{array}{l} TFP_{it}=\alpha +\beta_{1}Diver_{i}+\beta_{2}Perstu_{it}+\sum^{\text{​}}Controls_{it}+Year\;\\ +Industry+\varepsilon \end{array} \end{array} \end{array}$$

The *Perstu*_*it*_in formulas (8) and (9) represents the accumulation of human capital, as measured by the proportion of students in urban institutions of higher learning in the total population (Chen et al., 2013). Other variables are illustrated above.

Table 6 lists the results of the intermediary mechanism test of human capital accumulation. Columns (2) and (3) provide the estimation results of models (8) and (9), respectively. The effect of *Diver* on human capital accumulation was significantly negative in column (2). However, after controlling the intermediary variable human capital accumulation (*Perstu*), the absolute value of the Diver's coefficient was smaller in column (3), and the coefficient of *Perstu* on TFP was significantly positive, which indicates dialect diversity hindered human capital accumulation, and thus inhibited TFP of the enterprise. Therefore, **Hypothesis 3** holds.

**Table 6:** Results for mechanism analyses: human capital accumulation.

	**(1)**	**(2)**	**(3)**
	***TFP_ACF***	***Perstu***	***TFP_ACF***
*Diver*	−0.322***	−0.249***	−0.288***
	(0.074)	(0.019)	(0.074)
*Perstu*			0.482***
			(0.183)
*Controls*	Yes	Yes	Yes
*Year FE*	Yes	Yes	Yes
*Industry FE*	Yes	Yes	Yes
*N*	10,875	10,875	10,875
*Adj. R^2^*	0.407	0.264	0.408

This table presents the human capital accumulation mechanism results between dialect diversity and TFP. Perstu represents the accumulation of human capital, as measured by the proportion of students in urban institutions of higher learning in the total population. The definition of all other variables can be seen in section “Model specification and variable definition.” A series of fixed effects are also included and T-statistics are provided in parentheses.

The ^***^ symbol indicates the significance at the 1% level. Standard errors are clustered at the firm level.

#### Verification of innovation mechanism

The following mediation effect model was constructed to investigate whether dialect diversity can affect the TFP of enterprises through an innovation mechanism.


(10) $$\begin{array}{l} {TFP}_{it}=\alpha +\beta {Diver}_{i}+\sum {Controls}_{it}+Year+Industry+\varepsilon \end{array}$$

(11) $$\begin{array}{l} {LnRD}_{it}=\alpha +\beta {Diver}_{i}+\sum {Controls}_{it}+Year+Industry+\varepsilon \end{array}$$

(12) $$\begin{array}{l} \begin{array}{l} \begin{array}{l} TFP_{it}=\alpha +\beta_{1}Diver_{i}+\beta_{2}LnRD_{it}+\sum^{\text{​}}Controls_{it}+Year\;\\ +Industry+\varepsilon \end{array} \end{array} \end{array}$$

In formulas (11) and (12), *LnRD*_*it*_represents the logarithm of innovation input. Following Zhang and Wang (2022), the ratio of R and D investment and the total number of R and D employees indicate the innovation investment. Other variables are consistent, as described above.

Table 7 reports the results of the innovative mediation mechanism. Columns (2) and (3) provide the estimation results of models (11) and (12), respectively. We found that the coefficient of *Diver* was significantly negative in column (2). However, after adding intermediary variable innovation input (*LnRD*), the absolute value of the Diver's coefficient was smaller, and the coefficient of *LnRD* on TFP was significantly positive, indicating that dialect diversity reduces enterprise innovation input and inhibits enterprise TFP. Hence, **Hypothesis 4** holds.

**Table 7:** Results for mechanism analyses: innovation.

	**(1)**	**(2)**	**(3)**
	***TFP_ACF***	***LnRD***	***TFP_ACF***
*Diver*	−0.322***	−0.432**	−0.319***
	(0.074)	(0.173)	(0.074)
*LnRD*			0.005*
			(0.003)
*Controls*	Yes	Yes	Yes
*Year FE*	Yes	Yes	Yes
*Industry FE*	Yes	Yes	Yes
*N*	10,875	10,875	10,875
*Adj. R^2^*	0.407	0.638	0.407

This table presents the innovation mechanism results between dialect diversity and TFP. LnRD represents the logarithm of innovation input. as measured by the ratio of RandD investment and the total number of RandD employees. The definition of all other variables can be seen in section “Model specification and variable definition.” A series of fixed effects are also included and T-statistics are provided in parentheses.

^***^, ^**^, and ^*^ indicate significance at the 1, 5, and 10% levels, respectively. Standard errors are clustered at the firm level.

### Cross-section analysis

#### Region-based difference analysis

China has vast lands and abundant resources, separating the northern and southern regions, thus forming different cultures, and its cultural differences are also reflected in the dialects. The formation of dialects is closely related to the natural geographical barrier. The north has few rivers and mountains, the terrain is flat, and the difference between different local dialects is slight. Most of the northern dialects are also called “Mandarin.” In the south, mountains, rivers, lakes, and seas abound, and the terrain is undulating and changeable. As a result, considerable differences between dialects can be observed, making communication between dialects challenging. Therefore, if cultural diversity inhibits enterprises' TFP, it can be expected that compared with the northern region, the complex language environment of the southern region will make the factor flow more difficult, reduce innovation investment, and further inhibit the TFP of enterprises.

To test this conjecture, we refer to Liu et al. (2019) to set the south of the Yangtze River as the southern region and the north of the Yangtze River as the northern region. The regression results are shown in Panel A of Table 8, and the regression coefficient of *Diver* was only significant in the southern region, indicating that dialect diversification's inhibitory effect on enterprises' TFP was more significant in the southern region. The views of this paper are, therefore, verified.

**Table 8:** Results of the effect of dialect diversity on TFP in different subsamples.

**Panel A: Northern region vs. southern region**		
	**North**	**South**
	**(1)**	**(2)**
*Diver*	0.013	−0.475***
	(0.124)	(0.110)
*Control*	Yes	Yes
*Year FE*	Yes	Yes
*Industry FE*	Yes	Yes
*N*	4,825	5,065
*Adj. R^2^*	0.431	0.422
**Panel B: high-tech industry vs. non-high-tech industry**		
	**High-tech**	**Non-high-tech**
	**(1)**	**(2)**
*Diver*	−0.219**	−0.386***
	(0.086)	(0.132)
*Control*	Yes	Yes
*Year FE*	Yes	Yes
*Industry FE*	Yes	Yes
*N*	5,479	4,388
*Adj. R^2^*	0.263	0.461
**Panel C: SOEs vs. non-SOEs**		
	**Non-SOEs**	**SOEs**
	**(1)**	**(2)**
*Diver*	−0.222**	−0.380***
	(0.089)	(0.118)
*Control*	Yes	Yes
*Year FE*	Yes	Yes
*Industry FE*	Yes	Yes
*N*	4,823	5,158
*Adj. R^2^*	0.379	0.439
**Panel D: capital-intensive vs. non-capital-intensive**		
**enterprises**		
	**Non-capital intensive**	**Capital-intensive**
	**(1)**	**(2)**
*Diver*	−0.474***	−0.163
	(0.115)	(0.104)
*Control*	Yes	Yes
*Year FE*	Yes	Yes
*Industry FE*	Yes	Yes
*N*	4,521	4,448
*Adj. R^2^*	0.331	0.424
	**(1)**	**(2)**
*Diver*	−0.414***	−0.139
	(0.093)	(0.088)
*Control*	Yes	Yes
*Year FE*	Yes	Yes
*Industry FE*	Yes	Yes
*N*	4,188	6,053
*Adj. R^2^*	0.417	0.408

This table reports the baseline regression results of estimating the impact of dialect diversity on TFP in different subsamples. In Panel A, B, C, D, and E, we used region, industry, corporate ownership types, capital intensity, and social trust to group the subsamples, respectively. The definition of all other variables can be seen in section “Model specification and variable definition.” A series of fixed effects are also included, and T-statistics are provided in parentheses.

^***^ and ^**^ indicate significance at the 1 and 5% levels, respectively. Standard errors are clustered at the firm level.

#### Industry-based difference analysis

Technology makes several ambiguous arguments. In general, when a merger takes place between two high-tech firms, the management or employees communicate using concise and clear technology words. Thus, the high-tech industry is less influenced by cultural differences. We tested this conjecture by dividing the sample between high-tech and non-high-tech industries. We expected to find that the effect of linguistic distance, one aspect of cultural difference, would be weaker in the high-tech industry (Li et al., 2018).

Panel B of Table 8 presents the regression results for high-tech and non-high-tech industries. All control variables are the same as those in Table 2. The coefficient of *Diver* was more significant for the non-high-tech industry, suggesting that language friction plays a more significant role in industries such as manufacturers and retailers.

#### Property rights-based difference analysis

State-owned enterprises (SOEs) and non-state-owned enterprises (non-SOEs) have specific differences in financing constraints and internal control (Johnson et al., 2002). Whether this difference affects the relationship between dialect diversification and TFP was one of the issues explored in this paper. Therefore, we grouped the samples according to SOEs and non-SOEs.

Panel C of Table 8 presents the regression results for non-SOEs and SOEs, respectively. The Table indicates that the regression results for SOEs were negative and significant at a 1% level. At the same time, the coefficients for the non-SOEs were also positive but much smaller and more insignificant in the measurement of TFP, indicating that dialect diversification on TFP plays a more significant role for SOEs.

Specifically, SOEs have the social responsibility to address employment issues, and the flow of employees is poor, making it easier for them to group small groups, which increases the negative effects of dialect diversification on the TFP of enterprises; however, the high employee turnover rate of non-SOEs suppresses the identity effect of dialects. Meanwhile, the labor force flow brings advanced knowledge and technology to enterprises and weakens the inhibitory effect of dialect diversification on the TFP of non-SOEs.

#### Capital intensity-based difference analysis

Compared with labor-intensive enterprises, capital-intensive enterprises tend to have a greater demand for advanced technology and talents and focus more on the quality of the labor force. Therefore, the specialized knowledge and skills of employees in this industry have a decisive effect on enterprise productivity (Kahn and Lim, 1998; Lepak et al., 2003), while dialect diversification suppresses enterprise capital flow and human capital accumulation, thus suppressing enterprise TFP.

On the one hand, the dialects in different regions vary greatly, and communication barriers are formed between the regions, which reduce the flow and allocation of capital, hinder the capital accumulation of the regions, and inhibit enterprise TFP. On the other hand, dialect diversification affects the flow of people between regions, which is not conducive to the accumulation of human capital of enterprises, hinders the introduction of advanced talents, and suppresses the TFP of enterprises. Therefore, this paper expected high capital-intensive enterprises to be more vulnerable to dialect diversification's inhibitory effect on enterprise TFP. This paper, referring to Li and Sheng (2019), measured the capital intensity of enterprises with the natural logarithm of fixed net asset value and the number of employees and grouped the samples according to the median capital intensity. The enterprises above the median are capital-intensive enterprises, and those below the median are set as labor-intensive enterprises.

The regression results are shown in Panel D in Table 8. The regression coefficient of *Diver* was only significant in capital-intensive industries, indicating that dialect diversification has a more significant inhibitory effect on TFP in capital-intensive industries. The views of this paper were verified.

#### Social trust-based difference analysis

The external environment may influence innovation and factor mobility of enterprises. Specifically, we argue that social trust may significantly affect firms' innovation and factor mobility. First, social trust affects the innovation of enterprises (Ajzen, 1985). Research in sociology shows that social trust enables people to form an emotional relationship with one another and to reduce the distance in their interpersonal communication (Giddens, 1990). When the level of social trust (Van Lange et al., 1998; Yang and Farn, 2009) is high, individuals are more inclined to engage in altruistic behavior and share resources with others. Therefore, improving social trust can reduce cliquish behavior, reduce communication costs, promote cooperative behavior, and enhance corporate innovation, thereby reducing dialect diversity's negative effect on firms' TFP. Second, social trust affects the factor flow of enterprises. In a higher social trust environment, actors have excellent and stable expectations for the counterparties, which reduces transaction uncertainty, decreases information asymmetry and transaction costs, reduces waste and mismatch of resources, improves the efficiency of resource utilization, and reduces the negative effects of dialect diversity on the TFP of enterprises.

Referring to Li et al. (2019) and Liu and Li (2019), we used the Social Trust Index of each province obtained from the Chinese General Social Survey to measure social trust. The samples were also grouped by the median of the provincial social trust index. Panel E of Table 8 displays the regression results. The regression results in column (1) indicate that regional cultural diversity significantly and negatively correlates with corporate philanthropy in regions with lower social trust. In column (2), however, the coefficients of *Diver* are insignificant. The combined results indicate that higher social trust weakens the negative relationship between dialect diversity and TFP by increasing innovation and factor flow.

## Conclusion

In this study, we examined whether and how dialect diversity affects the TFP of listed companies from the perspective of psychological distance. Unlike previous studies, we investigated the effect of informal institutions such as dialect diversity on TFP (e.g., Feng et al., 2021; Fu and Zhang, 2022; Wang et al., 2022). Using all Chinese-listed firms from 2007 to 2019, we found that dialect diversity significantly inhibits a firm's TFP. This conclusion still holds after considering the endogenous problem and redefining the variables. Further research indicated that this negative effect was more pronounced for southern firms, capital-intensive firms, state-owned enterprises, and firms in non-high-tech industries. The results of the mechanism test indicated that factor mobility, human capital accumulation, and innovation were three critical ways dialect diversity affects firm innovation.

The findings of this study have some policy implications. First, policy formulation should consider the effects of informal institutions. This paper finds that dialect significantly suppresses TFP. Informal systems can sometimes play a more significant social role than formal systems, especially in countries where formal systems are not yet in place (Liu et al., 2022). If the influence of informal institutions is ignored in the policy formulation process, it may lead to actual policy effects deviating from expectations. Second, cultural barriers should be removed to improve the level of trust. We found that dialect diversity created cultural segmentation, increased transaction costs, and reduced trust levels, impeding factor mobility, human capital accumulation, and innovation, significantly inhibiting firms' TFP. Therefore, while protecting regional cultural diversity, cultural barriers between different regions should be actively eliminated to improve the level of trust, achieve optimal allocation of factors, and reduce the harm of dialects on TFP.

We identified some limitations of our study and discussed directions for future research. First, more sample data are needed for our study. Missing data from remote areas may have had specific effects on the regression results. Second, transmission channel issues must be addressed because many factors affect TFP. Finally, future studies could explore the linkage between dialects and TFP when more data become available.

## Data availability statement

The original contributions presented in the study are included in the article/supplementary material, further inquiries can be directed to the corresponding author.

## Author contributions

All authors listed have made a substantial, direct, and intellectual contribution to the work and approved it for publication.

## Funding

We gratefully acknowledges the financial support from National Natural Science Foundation of China (Grant Nos. 72162019 and 72102010), Social Science Fund of Jiangxi Province (Grant No. 21YJ30).

## Conflict of interest

The authors declare that the research was conducted in the absence of any commercial or financial relationships that could be construed as a potential conflict of interest.

## Publisher's note

All claims expressed in this article are solely those of the authors and do not necessarily represent those of their affiliated organizations, or those of the publisher, the editors and the reviewers. Any product that may be evaluated in this article, or claim that may be made by its manufacturer, is not guaranteed or endorsed by the publisher.
